# A Novel Method for Identifying Frailty and Quantifying Muscle Strength Using the Six-Minute Walking Test

**DOI:** 10.3390/s24144489

**Published:** 2024-07-11

**Authors:** Yunjin Zhang, Minoru Morita, Tsunahiko Hirano, Keiko Doi, Xin Han, Kazuto Matsunaga, Zhongwei Jiang

**Affiliations:** 1 Faculty of Engineering, Graduate School of Sciences and Technology for Innovation, Yamaguchi University, 2-16-1 Tokiwadai, Ube 755-8611, Japan; 2 Department of Respiratory Medicine and Infectious Disease, Graduate School of Medicine, Yamaguchi University, 1-1-1, Minamikogushi, Ube 755-8505, Japan; 3 Department of Pulmonology and Gerontology, Graduate School of Medicine, Yamaguchi University, 1-1-1, Minamikogushi, Ube 755-8505, Japan

**Keywords:** six-minute walking test, frailty, sarcopenia, walking muscle strength, stride length, step cadence

## Abstract

The six-minute walking test (6MWT) is an essential test for evaluating exercise tolerance in many respiratory and cardiovascular diseases. Frailty and sarcopenia can cause rapid aging of the cardiovascular system in elderly people. Early detection and evaluation of frailty and sarcopenia are crucial for determining the treatment method. We aimed to develop a wearable measuring system for the 6MWT and propose a method for identifying frailty and quantifying walking muscle strength (WMS). In this study, 60 elderly participants were asked to wear accelerometers behind their left and right ankles during the 6MWT. The gait data were collected by a computer or smartphone. We proposed a method for analyzing walking performance using the stride length (SL) and step cadence (SC) instead of gait speed directly. Four regions (Range I–IV) were divided by cutoff values of SC = 2.0 [step/s] and SL = 0.6 [m/step] for a quick view of the frail state. There were 62.5% of frail individuals distributed in Range III and 72.4% of non-frail individuals in Range I. A concept of a WMS score was proposed for estimating WMS quantitatively. We found that 62.5% of frail individuals were scored as WMS1 and 41.4% of the non-frail elderly as WMS4. The average walking distances corresponding to WMS1–4 were 207 m, 370 m, 432 m, and 462 m, respectively. The WMS score may be a useful tool for quantitatively estimating sarcopenia or frailty due to reduced cardiopulmonary function.

## 1. Introduction

Muscles play vital roles in maintaining life and health; can perform various actions based on our own subjective will; and enable us to move, maintain balance, consume energy, etc. [1]. However, with age and physiological changes, muscle mass and strength gradually decline, leading to limitations in basic physical activities for older adults, such as walking, climbing stairs, and lifting things [2]. These limitations negatively impact daily life and may further lead to age-related diseases such as sarcopenia and frailty [3,4]. Therefore, muscle function is regarded as a key indicator for exploring the aging process and evaluating individual health [5,6].

The main physical assessment indicators for muscle function are handgrip strength and walking speed [7,8,9]. These indicators have been well-validated and are easy to implement in clinical practice, representing the strength of the upper and lower limb muscles. Additionally, they have been incorporated into the revised Japanese version of Cardiovascular Health Study (J-CHS) [10,11] for assessing frailty. Frailty is a clinical status characterized by physical, psychological, and social vulnerability [12,13]. The J-CHS includes two physical items (handgrip strength and walking speed) and three questionnaire items (unconscious weight loss, self-reported fatigue, and decreased physical activity), totaling five items to assess frail, pre-frail, or non-frail.

Existing studies [14,15] indicate that more than half of frail cases are due to physical causes such as sarcopenia, and the remaining are attributed to mental or social effects. Although the intrinsic connection among the five clinical manifestations remains unclear, physical frailty often has irreversible consequences compared to mental frailty [16]. Physical frailty directly leads to mobility impairment, making individuals more vulnerable to injury and potentially increasing the risk of illness and disability in the elderly. Mental frailty mainly manifests in mental health and social contact, with less pronounced effects on daily life and health compared to physical. Currently, a significant challenge with the J-CHS method is its inability to accurately identify physical frailty resulting from a decline in muscle function; also, it cannot provide a detailed quantitative description of the muscle capacity. Furthermore, the J-CHS model is a semi-objective assessment method that heavily relies on survey questionnaires, usually requiring the elderly to visit medical institutions. This approach fails to meet the needs of continuous care for the elderly and adds an additional burden onto informal caregivers.

To recognize physical frailty and assess muscular function more accurately, it is necessary to focus on physical activities [17,18]. Some studies, such as Toosizadeh et al.’s upper limb elbow flexion exercise [19], Bortone et al.’s gait analysis [20], and Greene et al.’s timed up and go (TUG) [21] test, have demonstrated certain feasibility in frailty assessment, yet they do not elucidate the muscle status. It is reported that moderate physical activity positively influences muscle contraction strength and immune cell function [22]. Walking is the easiest form of physical activity and is available to everyone who has no specific disease [23]. The decline in walking function can lead to muscle-related diseases [24]. Thus, we believe that monitoring walking activity can provide a better understanding of muscle health and early intervention in the onset of frailty.

Walking is a representative aerobic exercise that can improve the contractile function of lower limb muscles and cardiorespiratory endurance. A stable gait is an essential requirement for various activities and independent living [25]. Gait speed is the most important parameter representing lower limb muscle capability and is widely used to identify health risks and prevent chronic diseases [26]. Lim et al. suggested that gait speed has a significant impact on muscle fiber length and the ground reaction force of muscles by biomechanical analyses [27].

According to our knowledge, gait speed can be subdivided into stride length (SL) and step cadence (SC) [27,28]. However, no consensus has been reached regarding the relationship between SL and SC with muscle function in older people. A report of runners found that SL was significantly and positively correlated with the slow-speed group but was not significantly correlated with the fast-speed group [29]. We speculate that SL may reflect muscle extensibility and be related to the state of the slow-twitch fibers that represent persistent and low-intensity exercise. In addition, our investigations showed that athletes’ changes in SC increased significantly and exceeded the surpassing changes in SL during uniform and sprint running [29]. This suggests that SC may represent the state of fast-twitch fibers that generate instantaneous force in muscles. Therefore, we speculated that using SL and SC instead of gait speed may provide a more detailed assessment of sarcopenia and physical frailty caused by lower limb muscle weakness.

The six-minute walking test (6MWT) is a standardized clinical assessment method of physical ability by the American Thoracic Society (ATS) [30]. It is widely used in clinical practice to assess exercise endurance and respiratory-related diseases, and the walking distance (6MWD) is considered an indicator of good cardiopulmonary function and outdoor activity ability. In Japan, the 6MWT is implemented at hospitals or special facilities and is covered by medical insurance [31] but still faces a shortage of personnel. In recent years, the widespread use of sensor technology has facilitated an easier and simpler testing process [32].

Our survey found that only a few studies have used the 6MWT as a gait task to assess frail or muscle status in the elderly, and the distance in 6MWT is the only index to estimate body movement ability. This study aims to develop a wearable measuring system for the 6MWT and proposes a novel method for identifying frailty and quantifying muscle strength. We analyze the parameters obtained by the 6MWT and compare them to the assessment results of the J-CHS criteria to explore a method for assessing and visualizing sarcopenia frailty. In addition, the concept of a WMS score is proposed for estimating the walking muscle strength quantitatively. To verify the validity of the WMS score, we also investigated its correlation with the indicators of the 6MWD and handgrip strength. The proposed method in this study may provide an objective and effective method for the early diagnosis and health management of the elderly.

The acronyms used in this manuscript can be found in Table A1 in Appendix A.

The following are the main contributions of this study:

(1) A measurement and analysis system for the 6MWT using wearable accelerometers was developed.
(2) A method for discrimination of the frailty and visualization of lower limb muscle weakness based on the gait parameters of SL and SC was provided.
(3) We proposed the concept of a WMS scale as a multivariate function of SL, SC, and six-minute walking energy expenditure (6MWEE) for scoring the walking muscle strength.

## 2. Materials and Methods

### 2.1. Participants

We recruited 60 elderly participants (46 males and 14 females, age 72 ± 5 years, weight 63 ± 12 kg, height 163 ± 8 cm) from August 2021 to July 2022 at the university-affiliated hospital as our study sample. These participants had basic physical activity capabilities and no diseases that would affect their ability to complete the assessments in this study. Before the experiment, the research staff explained the study protocol to the participants and obtained their written informed consent. This research project was approved by the local (Ube, Japan) institutional review board (No. H2021-031).

The demographic characteristics and frailty status of the participants, as shown in Table 1, indicate that 8 (13.4%) participants were in the frail condition, 23 (38.3%) participants were in the pre-frail condition, and 29 (48.3%) participants were in the non-frail condition.

### 2.2. J-CHS for Frailty Assessment

The clinical diagnosis of frailty in Japan generally uses the J-CHS criteria [10], as shown in Table 2. The first three items were assessed by questionnaires, and the other two items were assessed by clinical tests. The handgrip strength (HS) was measured using a Digital Grip Dynamometer (Takei Scientific Instruments Co., Ltd., Niigata, Japan) as follows. The participants were instructed to stand upright, let their arm hang naturally, and clasp the grip with full force. Two readings were obtained from the right and left hands, and the average value was used as the measured HS value for each participant. Walking speed was obtained through the five-meter walking test, with a cutoff value of 1 m/s for both males and females. According to the J-CHS criteria, each item is scored as one point, with a total score of *n* = 5. The score of *n* ≥ 3 is defined as frail, the score of *n* = 1–2 is as pre-frail, and the score of *n* = 0 is defined as non-frail.

### 2.3. Gait Measurement System (GMS)

We installed two TWELITE 2525A (Mono Wireless Inc., Kanagawa, Japan) wearable accelerometer sensors (dimensions: 20 × 20 × 10 mm, weight: 6.5 g, sampling rate: 50 Hz) on the posterior ankles of the participants and constructed a gait measurement system (GMS) for collecting gait data.

**Figure 1:**
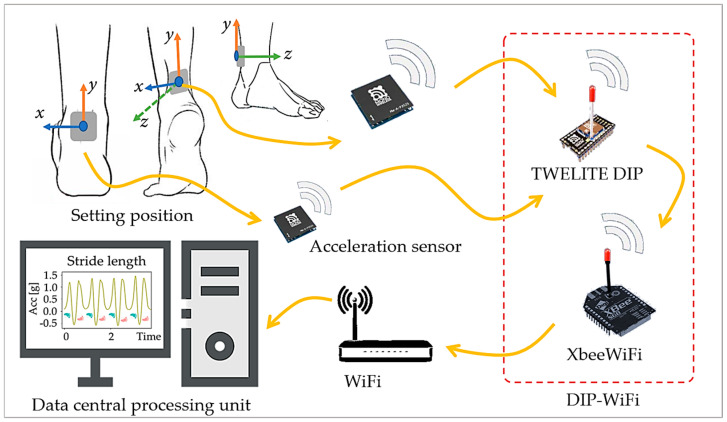
Schematic diagram of the wearable accelerometer’s setting position and gait measurement system (Up direction: forward direction of the *y*-axis. Walking direction: forward direction of the *z*-axis. Left direction: forward direction of the *x*-axis).

The GMS consists of two wireless accelerometer sensors, a DIP-WIFI signal repeater, a WiFi router, and a data analysis and management server. As shown in Figure 1, the accelerometer sensors transmit the collected gait data wirelessly to the signal repeater. The repeater receives the signal via TWELITE DIP and sends the data from the XbeeWiFi device to the data storage center through serial communication. This GMS has the advantages of a wide measurement range and a high signal reception success rate (>95%).

### 2.4. Six-Minute Walking Test (6MWT)

**Figure 2:**
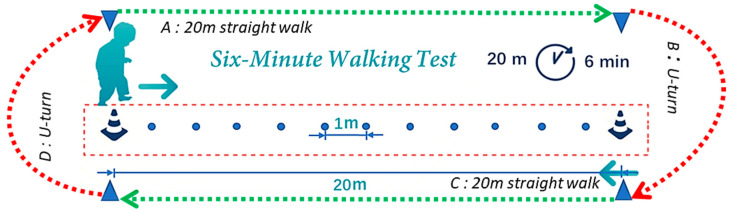
Schematic diagram of the 6MWT. The participants started from the cone-shaped barrel on the left and walked along experimental routes A, B, C, and D at their fastest speed for 6 min. There are some marking points between the two cone-shaped barrels with an interval of 1 m.

We monitored the gait data during the 6MWT using the GMS. The experimental design [30] required participants to walk back and forth as quickly as possible for 6 min in the corridor, as shown in Figure 2. A corridor with a straight line length of 20 m or more was used to set up the 6MWT course.

### 2.5. Preprocessing and Gait Parameters

#### 2.5.1. Data Preprocessing

During the test, the collected data from the sensors included both gravitational acceleration and the participant’s walking acceleration. To obtain the actual acceleration data, we needed to separate the gravitational acceleration.

For each component of the three-dimensional sensor, a low-pass filter was employed to isolate the gravitational acceleration from the total acceleration. This separated gravitational acceleration was then subtracted from the total acceleration to derive the gait acceleration [33]. We preprocessed the data using an infinite impulse response (IIR) filter, as shown in Equation (1).

(1) $$x_{g}(t)=\alpha_{0}(x\left(t\right)\beta_{0}+x\left(t-1\right)\beta_{1}+x\left(t-2\right)\beta_{2}-x_{g}(t-1)\alpha_{1}-x_{g}(t-2)\alpha_{2})$$

In Equation (1), $\alpha_{0},\;\beta_{0},\;\beta_{1},\;\beta_{2},\;\alpha_{1}$, and $\alpha_{2}$ were the filter coefficients. We computed these coefficients using a second-order Butterworth low-pass filter as in Equation (2), where the normalized cutoff frequency was calculated as $w_{c}=2f_{c}/f_{s}$ (with a cutoff frequency $f_{c}=0.2\;\left[\mathrm{Hz}\right]$ and a sampling frequency $f_{s}=50\;\left[\mathrm{Hz}\right]$).

(2) $$\left\{\begin{array}{c} \begin{array}{c} \alpha_{0}=1\\ \alpha_{1}=\frac{2\left(1-w_{c}^{2}\right)}{w_{c}^{2}}\\ \alpha_{2}=\frac{\left(\sqrt{2}w_{c}-w_{c}^{2}-1\right)}{w_{c}^{2}} \end{array}\\ \begin{array}{c} \beta_{0}=1\\ \beta_{1}=2\\ \beta_{2}=1 \end{array} \end{array}\right.$$

To enhance the accuracy and reliability of the gait acceleration data, we used a Kalman filter to predict the walking state, aiming to eliminate potential errors and noise in the data [34].

The GMS only measures acceleration data, while walking was a dynamic system evolving over time, including information such as acceleration, velocity, and position. Here, the state variable $x_{t}$ in Equation (3) expresses the walking state in the x direction (similarly in the y and z directions).

(3) $$x_{t}=\left[\begin{array}{c} x\\ v\\ a \end{array}\right],\;(v=\dot{x},\;a=\ddot{x})$$

To obtain more accurate acceleration data, we used the Kalman filtering method described by Zarchan et al. [35], as shown in Equation (4).

(4) $$x_{t+1}=Fx_{t}+Gu_{t}+w$$

where F is the state transition matrix, G is the control input matrix, and the parameters are set in the program as shown in Equation (5). $u_{t}$ is the acceleration data in the x, y, and z directions, respectively. w is the process noise, assumed to be Gaussian with covariance. The initial settings for foot position, velocity, and acceleration were all set to 0, and $dt=1/f_{s}=1/50$.

(5) $$F=\left[\begin{array}{ccc} 1 & dt & \frac{1}{2}dt^{2}\\ 0 & 1 & dt\\ 0 & 0 & 1 \end{array}\right],\;G=\left[\begin{array}{c} \frac{1}{2}dt^{2}\\ dt\\ 1 \end{array}\right]$$

**Figure 3:**
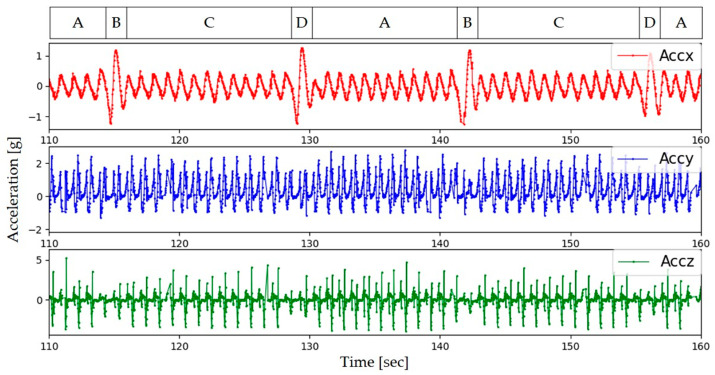
A portion of the acceleration data after signal processing. Where A and C are the straight walk parts, and B and D are the U-turn walk parts. The *x*-axis (left-right direction) data intuitively represent the difference between the two walking parts.

Figure 3 shows a portion of the time waveform of the acceleration data (x, y, and z) that have been preprocessed by Equations (1) and (4). According to the guidelines of the 6MWT (Figure 2), we divided the data into four parts, namely the straight walk (SW) parts A and C and the U-turn walk (UW) parts B and D. When making a UW, people tend to have a deceleration process, due to a subconscious self-protective mechanism. Therefore, in this study, the walking acceleration data during the SW parts were used for gait analysis.

Since the x-direction accelerometer corresponds to the left–right direction of the subject, the motion amplitude in the *x*-axis becomes larger than that in the straight part during the UW. Figure 3 intuitively demonstrates the difference between the SW and UW in the x-direction, which is the basis for segmenting the gait data of the SWs from the UWs.

#### 2.5.2. Calculation of Gait Parameters

Five gait parameters, namely stride length (SL), step cadence (SC), gait velocity (GV), six-minute walking distance (6MWD), and six-minute walking energy expenditure (6MWEE), were extracted and calculated as follows.

1. Stride Length (SL)

**Figure 4:**
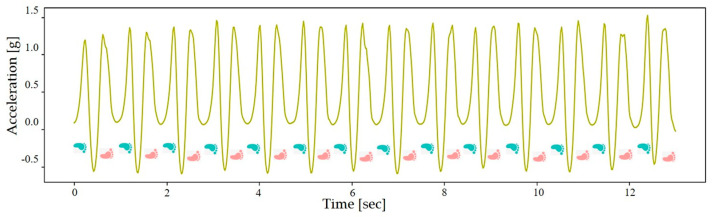
Analysis results of *y*-axis (up-down direction) acceleration data for straight walking.

SL [m/step] is the length during one action of lifting and placing the same foot [27]. Figure 4 shows the results of the kinematic analysis of the acceleration data in the up–down direction during each SW. Since the distance of each SW part is 20 m, we only needed to determine the number of steps to calculate SL by Equation (6).

(6) $$SL=\frac{20m}{step}$$

2. Step Cadence (SC)

**Figure 5:**
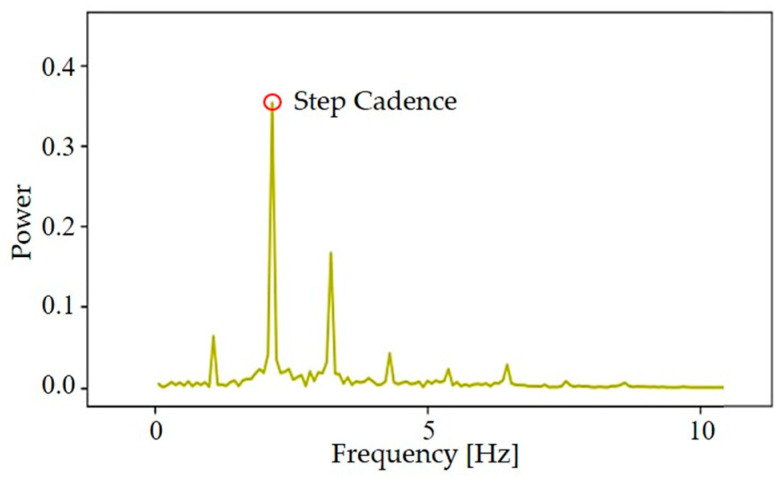
FFT analysis of the *y*-axis SW data (up–down direction). The average step cadence is the frequency value corresponding to the maximum peak.

SC [step/s] is the beat of walking and the number of steps per second [27]. We applied Fast Fourier Transform (FFT) to the acceleration data of each SW part and obtained the frequency component in Figure 5. In the spectrum diagram, the frequency component corresponding to the maximum amplitude represents the mean SC of this SW.

3. Gait Velocity (GV)

GV [m/s] is the product of SL and SC [27], as shown in Equation (7). *SL* and *SC* are the averages of the SW parts.

(7) GV=SL∗SC

4. Six-Minute Walking Distance (6MWD)

According to the 6MWT test guidelines, 6MWD [m] is the sum of all the SW parts’ distances in 6 min. Our calculation method is to add the distance of the complete SW parts, plus the final estimated partial SW distance, as shown in Equation (8).

(8) 6MWD=20∗p+SL∗q

where p is the number of complete SW, *SL* is the mean value calculated by Equation (7), and q is the number of steps recognized in the final partial SW.

5. Six-Minute Walking Energy Expenditure (6MWEE)

6MWEE [kcal/h] represents the total energy expenditure of the walking activity [36,37], which can be calculated by Equation (9). This calculation involves the individual’s basal metabolic rate (*BMR*), the metabolic equivalent of the task (*MET*), and the duration of the activity (*Time*).

(9) 6MWEE=BMR∗MET∗Time

BMR is the number of calories the body needs to perform basic life-sustaining functions [37]. The most widely used prediction equation for estimating the BMR is the Harris–Benedict equation (HBE) [38], which was developed in 1918, as shown in Equation (10). The HBE considers factors such as age (*A*), gender (*G*), weight (*W*: kg), and height (*H*: cm) to estimate the number of calories expended by an individual at rest.

(10) $$\left\{\begin{array}{c} Male:\;BMR=66.4730+13.7516*W+5.0033*H-6.7550*A\\ Female:\;BMR=655.0955+9.5634*W+1.8496*H-4.6756*A \end{array}\right.$$

MET is a unit used to estimate the amount of oxygen consumed and the number of calories burned during physical activity [39]. It represents the ratio of the metabolic rate during a specific physical activity to the resting metabolic rate, where a higher MET value indicates greater activity intensity. We calculated the MET of walking using the method proposed by Brooks et al. [39], as shown in Equation (11).

(11) MET=0.832∗GV−0.016∗W−0.196∗G+1.034

where GV is the velocity calculated by Equation (7), W is the body weight [Kg], and G is the gender (1: male, 2: female).

### 2.6. Statistical Analysis

We used the Shapiro–Wilk test to check whether the variables obeyed the normal distribution. The Student’s *t*-test was used for the two groups’ data that conformed to a normal distribution, and the ANOVA analysis was used for the data that did not conform to a normal distribution. We compared differences in the gait parameters among clinical diagnostic results (frail, pre-frail, and non-frail) and calculated the *p*-values to determine the significance of these differences. For some statistical tests, the mean value and standard deviation were reported, and *p*-values less than 0.05 were considered to be statistically significant. In addition, we calculated Cohen’s d effect size (d-value) to supplement the assessment of the practical impact of these differences, where d = 0.2, 0.5, and 0.8 were considered small, medium, and large effects. Combining the significance and effect size, we explored the significance of the gait parameters in frailty.

We performed receiver operating characteristic (ROC) analyses on the basic gait parameters to explore their role in the frailty assessment. Among the independent predictor variables, an area under the curve (AUC) of the ROC analysis greater than 0.7 was considered statistically significant, and its corresponding threshold was often used to develop differentiation models.

In this study, we used Python (version 3.8.1) to develop the GMS, employing NumPy and Pandas for data processing; SciPy for conducting the *t*-tests, ANOVA analyses, and calculating the *p*-values; Statsmodels for computing Cohen’s d effect sizes; and Scikit-learn for performing ROC curves and logistic regression analyses. Additionally, we employed Matplotlib and Sea-born for data visualization.

## 3. Results

### 3.1. J-CHS Criteria and Gait Parameters

Table 3 shows the results of the statistical analysis of the five gait parameters for the frail, pre-frail, and non-frail groups. The *p*-values and d-values were also calculated for comparisons between the frail and pre-frail groups and between the pre-frail and non-frail groups. Analysis of the *p*-values and d-values revealed that all gait parameters showed significant differences between the frail and pre-frail groups, with SC showing the largest effect size (*p* < 0.001, d = 1.712). On the other hand, there were no significant differences between the pre-frail and non-frail groups for these parameters, except for SL (*p* = 0.015, d = 0.707) and 6MWD (*p* = 0.019, d = 0.672). These results indicated that the gait parameters, specifically SC and SL, played an important role in the identification of frailty.

Table 4 shows the results of the relationship between the parameters (SL and SC) and the five assessment items of the J-CHS criteria. The WL item had no significant difference in the SL or SC, while the SP item showed significant differences in both the SL (*p* < 0.001) and SC (*p* < 0.001). The HS and EX items were significantly correlated with the SL (*p* < 0.001, d = 1.129) and SC (*p* = 0.004, d = 0.952), respectively.

### 3.2. SC and SL Distribution Map

**Figure 6:**
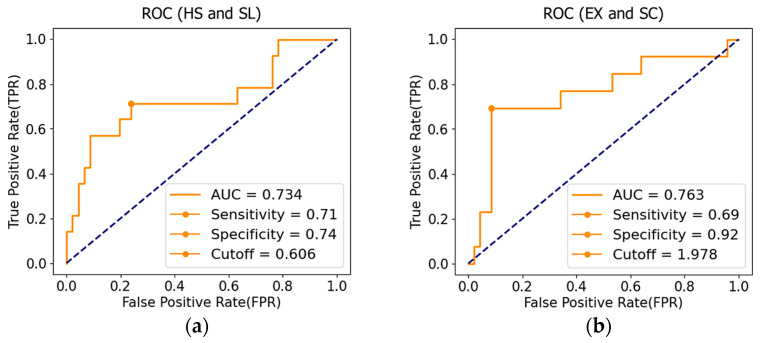
Cutoff values of SL and SC. (**a**) The ROC curve of SL versus HS (AUC is 0.734, the cutoff value is about 0.6 [m/step]); (**b**) The ROC curve to SC versus EX (AUC is 0.763, the cutoff value is about 2.0 [step/s]).

Figure 6a shows the ROC curve for SL versus the HS standard. The area under the curve (AUC) is 0.734, which is considered acceptable. The most distant point on the ROC curve corresponds to the classification threshold for the best performance (cutoff = 0.606 [m/step], sensitivity = 0.71, and specificity = 0.74). Figure 6b shows the ROC curve for SC versus the EX standard (AUC = 0.763, cutoff = 1.978 [step/s], sensitivity = 0.69, and specificity = 0.92). In the following analysis, the cutoff values of SL and SC are set at 0.6 [m/step] and 2.0 [step/s], respectively.

**Figure 7:**
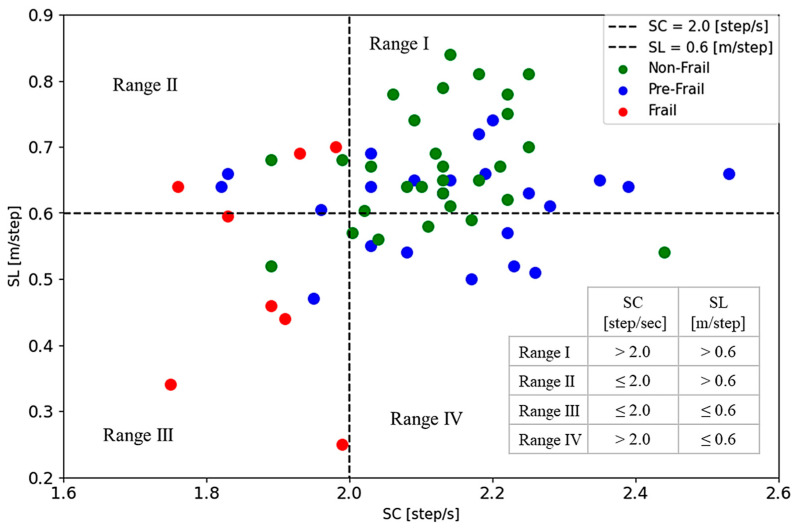
SC and SL distribution map. The dashed lines correspond to the threshold of 2.0 [step/s] for SC and 0.6 [m/step] for SL. The red marker is frail, blue marker is pre-frail, and green marker is non-frail, as assessed by the J-CHS.

**Figure 8:**
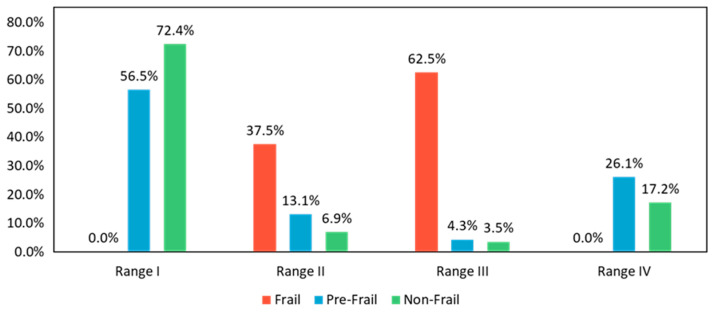
Percentages of the J-CHS assessment results in each of the four ranges.

Figure 7 shows a plot of the SC and SL distribution map to demonstrate the gait performance, where the horizontal axis represents SC and the vertical axis represents SL. The patients judged by the J-CHS criteria are described in different colors: frail in red, pre-frail in blue, and non-frail in green. The two dashed lines at SL = 0.6 [m/step] and SC = 2.0 [step/s] divide the map into four regions. Almost non-frail and some pre-frail cases are distributed in Range Ⅰ (SL > 0.6 [m/step], SC > 2.0 [step/s]), while almost frail cases are in Range Ⅲ (SL ≤ 0.6 [m/step], SC ≤ 2.0 [step/s]). Range Ⅱ (SL > 0.6 [m/step], SC ≤ 2.0 [step/s]) is a mixed region, and Range Ⅳ (SL ≤ 0.6 [m/step], SC > 2.0 [step/s]) shows mainly pre-frail with some of non-frail subjects. The percentage of each J-CHS assessment result in these four ranges is shown in Figure 8. There are 62.5% of the frail individuals concentrated in Range III and 72.4% of the non-frail individuals in Range I.

### 3.3. Concept of Walking Muscle Strength (WMS)

As described above, frailty is comprehensively caused by physical, psychological, and social factors. However, the 6MWT is a test only for checking physical functions. The results in Figure 7 and Figure 8 showed that the gait parameters (SL and SC) have a strong relationship with the J-CHS assessment results. This finding can be explained in that physical weakness or sarcopenia may be the most important factor causing frailty.

**Figure 9:**
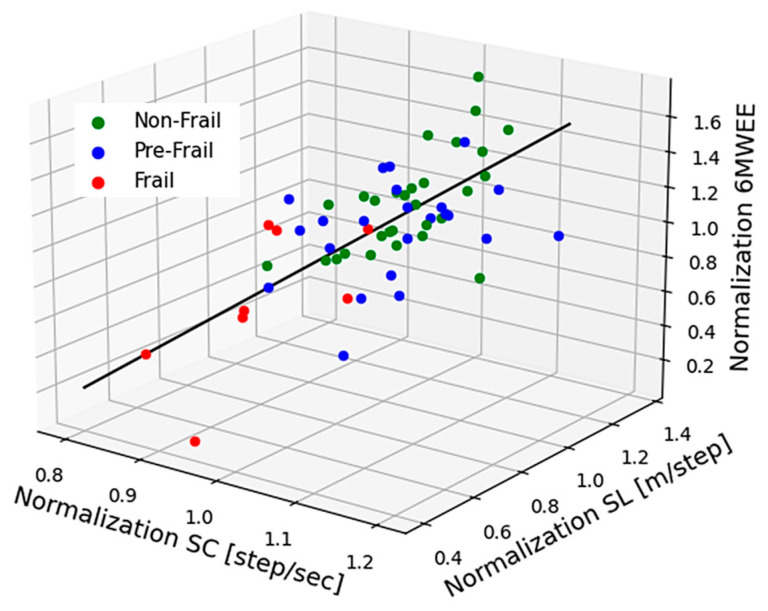
Three-dimensional scatter plot of SC, SL, and 6MWEE. The linear fit demonstrates the strong correlation (r = 0.853).

We define a variable WMS as a multivariate function of SL, SC, and 6MWEE for scoring muscle strength. 6MWEE is the energy expenditure [kcal/h] during the 6MWT, which is calculated from the MET for evaluating the level of physical activity in the elderly. Doctors typically develop different exercise programs based on the value of MET to improve the health status and quality of life for older people [40]. Figure 9 shows a three-dimensional scatter plot of SL, SC, and 6MWEE, in which each parameter was normalized by its mean value. It shows a strong correlation through linear fitting (r = 0.853). A WMS index is defined by the projection of each subject on the fitted line and is obtained by multiple regression analysis, as given by Equation (12).

(12) WMS=0.064∗SL+0.189∗SC+0.859∗6MWEE+1.554

**Figure 10:**
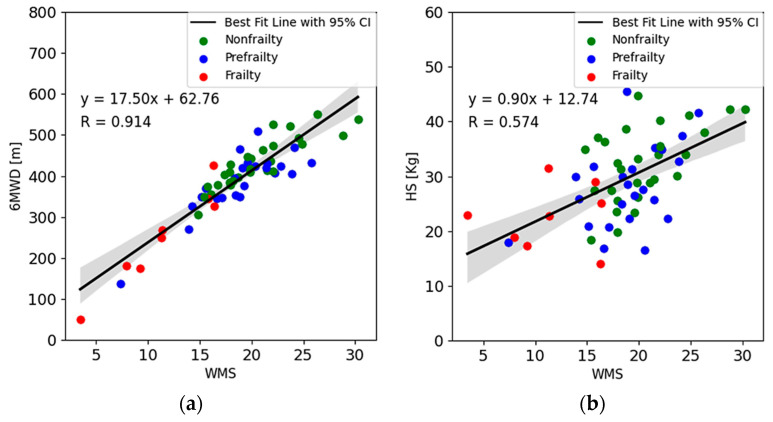
Correlations between the WMS index with physiological indicators: (**a**) 6MWD (r = 0.914) and (**b**) HS (r = 0.574).

To verify the validity of the WMS value obtained by Equation (12), a correlative analysis between the WMS index and other physiological indicators was conducted. The results are shown in Figure 10. According to clinical test guidelines [30], the 6MWD is an important parameter of the 6MWT, and Figure 10a shows a strong correlation (r = 0.914) between the 6MWD with WMS. Additionally, handgrip strength has been identified as an indicator of muscle strength [41]. The correlation of WMS with HS showed a moderate correlation (r = 0.574) in Figure 10b.

(13) $$S(WMS)=\frac{1}{1+e^{-a*(WMS-b)}}$$

**Figure 11:**
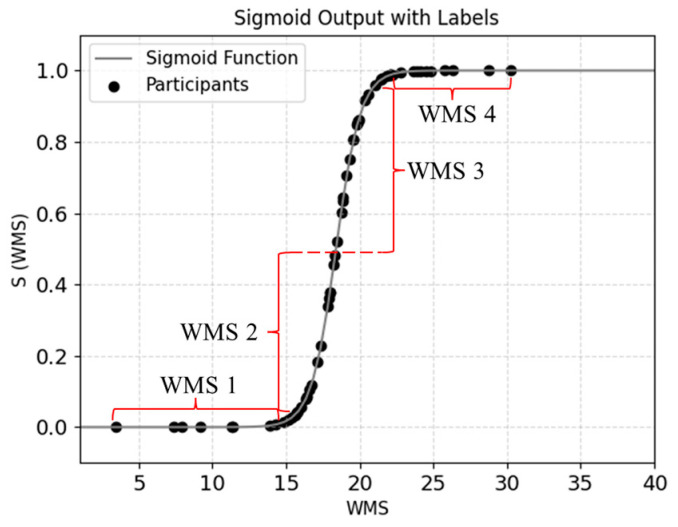
The WMS index of the participants was divided into four levels using the sigmoid function: WMS 1 (<0.01), WMS 2 (0.01–0.50), WMS 3 (0.50–0.95), and WMS 4 (>0.95).

To evaluate the physical walking performance using a simple scale, we normalized the WMS using the sigmoid function as given by Equation (13). Here, the parameters a and b were used to control the shape of the sigmoid function. In many medical studies and clinical evaluations, a 6MWD greater than 400 m was usually considered normal, reflecting good walking ability and a lower risk of cardiovascular disease [42]. The threshold of 400 m was used to label the WMS (6MWD ≥ 400 [m] as 1 and 6MWD < 400 [m] as 0), and the optimal parameters were obtained as a = 1.198 and b = 18.388 by the least squares method. Figure 11 shows the plot of WMS as a sigmoid function in the range between 0 and 1. Here, we defined a scale of WMS for scoring the walking muscle strength by WMS1 (<0.01), WMS2 (0.01–0.50), WMS3 (0.50–0.95), and WMS4 (>0.95).

**Figure 12:**
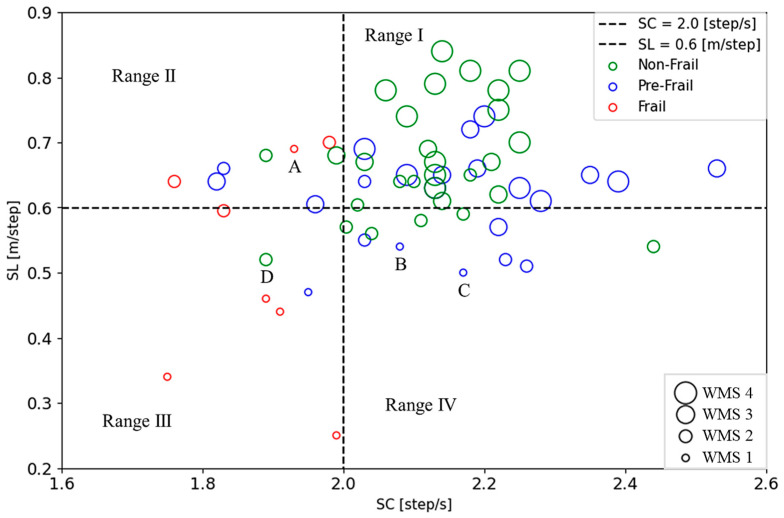
Replot of Figure 7 using the WMS scale. Marker size denotes muscle strength levels, and color indicates frail status by the J-CHS assessment (red as frail cases, blue as pre-frail cases, and green as non-frail cases).

Figure 12 replots Figure 7 using WMS scores. The size of the marker points represents the four levels of muscle strength, and the color represents the state of frailty based on the J-CHS assessment. WMS4s are mostly distributed in Range I, where the subjects are mainly non-frail and some pre-frail individuals. WMS1s are mainly in Range III, where the subjects are mainly frail with a few pre-frail and without non-frail individuals.

## 4. Discussion

### 4.1. Correlation between the J-CHS and Gait Parameters

The decline in GV of the elderly result is commonly regarded as a prominent feature of lower limb muscle failure and physical frailty [43]. Since GV is the coordinated result of SL and SC, we found that SL has a significant correlation with handgrip strength and SC has a correlation with exhaustion, as shown in Table 4. This finding aligns with our expectations that subjects with stronger upper limb handgrip strength also tended to have stronger lower limb strength [44,45], enabling them to take larger strides. Furthermore, muscle fatigue or mental exhaustion may lead to a slowing of pace. As assumed, these results suggest a close relationship between gait parameters and muscle function. SL may reflect muscle strength and extensibility associated with slow-twitch muscle fibers, while SC may be related to fast-twitch muscle fibers representing muscle fatigue.

The proposed gait parameters in Table 3 showed high sensitivity for identifying frail and non-frail individuals, especially the frail status due to muscle weakness. However, it seems more difficult to effectively discriminate the patients in pre-frail by these gait parameters. This is because the pre-frail individuals assessed by the J-CHS criteria are affected not only by physical frailty but also by psychological and social vulnerability.

### 4.2. SC and SL Distribution Map for Estimation and Visualization of Frailty

The proposed SC and SL distribution map is shown in Figure 7. Range Ⅰ indicates the muscle strength in a normal state, while Range Ⅲ represents much weaker muscle strength and easy fatigue. In Range III, both the fast-twitch and slow-twitch muscle fibers may be at their weakest state. Range Ⅱ and Range IV could be considered the slow-twitch or fast-twitch muscle fibers in a normal state, respectively.

In the current muscle impedance training, fast muscle fibers tend to be activated and developed through high-intensity and explosive exercises such as weightlifting and jumping, while slow muscle fibers are targeted for increased fiber thickness with light-load or low-intensity repetitive movements, such as walking and jogging [46]. For the elderly, the exercise of fast-twitch muscles is risky and impractical, so they may maintain and improve slow-twitch muscle fibers through low-intensity activities. This implies that patients in Range IV may recover to their normal state through physical activity, whereas those in Range Ⅱ may only maintain their current state or delay the decline to the Range III muscle state through appropriate intensity activity. Additionally, we also found that the average age of the four ranges gradually increases with the decline in muscle status (Range Ⅰ: 68.91 ± 6.79; Range Ⅳ: 73.00 ± 6.88; Range Ⅱ: 75.10 ± 6.66; Range Ⅲ: 76.67 ± 5.87). This also means that muscle decline is a common phenomenon among older people.

We compared the results of the J-CHS assessment in these four regions. The results in Figure 8 show that 62.5% of frail patients were in Range Ⅲ and 37.5% in Range II, while 72.4% of the non-frail patients were in Range Ⅰ and 17.2% in Range IV. These results suggest that the pre-frail and non-frail patients in Range II may develop sarcopenic frailty and that the pre-frail individuals in Range IV could return to a non-frail condition through appropriate intensity activity.

In addition, we found that about 56.5% of pre-frail cases were in Range I, who did not have a problem in the physical aspect. This fact might be subjectively influenced by the J-CHS questionnaire. Since this study was conducted during the COVID-19 pandemic, more than half of the pre-frail patients in Range I refrained from going out, which supports the validity of our results.

The SC and SL distribution map provides a practical and effective tool for a quick view of frail conditions in older people. This tool may enhance clinical assessment and aid in developing targeted interventions. For example, psychological guidance and regular physical activity may help patients in Range IV recover to a healthy state. Although the mechanisms underlying pre-frailty and the 6MWT are not fully understood, the 6MWT is mainly for evaluation on the physical aspects, and our proposed method may be more valuable and accurate in describing the sarcopenic state of the elderly.

### 4.3. Scoring the Walking Muscle Strength

We proposed the concept of scoring the WMS for the quantification of walking muscle strength. Four scales were defined from Equation (13) as WMS1 (<0.01), WMS2 (0.01–0.50), WMS3 (0.50–0.95), and WMS4 (>0.95). Compared to the J-CHS results, the WMS scores showed that 62.5% of frail and 13.1% of pre-frail patients scored WMS1, while 41.4% of non-frail and 30.4% of pre-frail patients scored WMS4. Furthermore, the average 6MWD values corresponding to WMS1–4 were 207 m, 370 m, 432 m, and 462 m, respectively. The 6MWD of all patients scored as WMS1 was lower than 330 m, which was the cutoff value for the survival rate [47] and high fall risk [48]. The 6MWD of all patients with a WMS4 score was higher than 400 m, which was the cutoff value to have a low risk of cardiovascular disease [42].

Looking at individual examples in Figure 12, patients A and B were both scored as WMS1 but belonged to different regions: Range II and Range IV, respectively. They were terminated from the experiment due to increased heart rate and shortness of breath during the 6MWT. Patient C’s score was WMS1, and they were diagnosed with sarcopenia (HS = 25.85 [kg], GV = 0.92 [m/s], and SMI = 5.5 [kg/m^2^]) based on the 2019 revised criteria of the Asian Sarcopenia Working Group (ASWG) [41]. Interestingly, patient D’s score as WMS2 was in Range III and was judged as non-frail by the J-CHS. This patient’s five-meter walking speed was 1.29 [m/s], which was higher than the J-CHS speed criteria. However, this patient’s walking speed of each 20 m in 6MWT varied greatly, and its average speed was 0.98 ± 0.19 [m/s] lower than the J-CHS standard. Additionally, patient D’s 6MWD was 306 m.

The WMS score introduced in this study provides a new method for quantifying muscle strength. Based on the above results and discussion, the proposed WMS scores and the SC and SL distribution map in Figure 12 may, with further research, become powerful tools for identifying sarcopenic frailty and guiding the rehabilitation of older people.

### 4.4. Advantages and Limitations

Compared to the other gait tasks, the 6MWT is characterized by a longer duration and the cognitive task involved (walking as long distance as possible), which can more effectively reflect the participants’ level of physical ability. Currently, medical interventions for frailty tend to be based on exercise therapy with nutritional support. The GMS for the 6MWT developed in this study provides convenience to healthcare providers and can also be used outside of clinical institutions, especially in home and outdoor environments.

Although the comprehensive gait parameters provide more detailed information about the health of the elderly, the current research also has certain limitations. First, the number of subjects, especially the frail patients, is relatively small, potentially limiting the generalizability of our findings. Second, stride length generally has some relationship with height, but we did not consider its effect in this study. For example, the participants in Range IV were shorter in height than those in the other regions. We also found that aging is also an important factor affecting stride length and step cadence, but this was not taken into consideration in this paper. Third, determining the WMS scores requires more clinical data collection and statistical analysis. Future research should involve larger and more diverse populations to validate the robustness of the SC and SL distribution map and WMS index across different demographic groups.

## 5. Conclusions

This study developed a measuring system with wearable accelerometers for the 6MWT and proposed a novel method for identifying frail states and quantifying muscle strength. Clinical data from 60 elderly participants revealed significant differences in the gait parameters of SL, SC, GV, 6MWD, and 6MWEE between the frail and non-frail groups. The SC and SL distribution map effectively visualized physical frailty and walking muscle weakness. Furthermore, walking muscle strength can be scored quantitatively using the proposed WMS scale, which is strongly correlated with the 6MWD and SC and SL distribution map. Future studies should include more participants and use more gait parameters to increase the sensitivity and reliability of the results. These findings from our study may provide more objective and accessible healthcare for older people.

## Figures and Tables

**Table 1:** Participant demographic characteristics and frailty assessment in the J-CHS.

Characteristic	Frail, *n* = 8 (13.4%)	Pre-Frail, *n* = 23 (38.3%)	Non-Frail, *n* = 29 (48.3%)
Male, (Female)	7, (1)	18, (5)	21, (8)
Age, years	78.38 ± 5.50	70.87 ± 8.02	70.21 ± 6.49
Height, cm	159.38 ± 7.22	163.87 ± 7.4	163.84 ± 8.06
Weight, kg	52.7 ± 12.42	67.27 ± 11.37	63.3 ± 10.71
Body mass index, kg/m^2^	20.64 ± 4.07	24.99 ± 3.67	23.64 ± 3.98
Handgrip strength, kg	22.65 ± 5.89	28.13 ± 7.62	32.42 ± 6.83
5-m walking speed, m/s	0.96 ± 0.30	1.25 ± 0.24	1.36 ± 0.18

**Table 2:** Japanese version of the frailty assessment criteria (J-CHS) [10].

Items	Assessment Criteria
(1) Weight Loss (WL)	Questionnaires: (Yes = 1, No = 0) Have you lost 2 kg or more in the past 6 months?
(2) Exhaustion (EX)	Questionnaires: (Yes = 1, No = 0) In the past 2 weeks, have you felt tired without a reason?
(3) Physical Activity (PA)	Questionnaires: (“No” to both questions = 1, others = 0) Do you engage in moderate levels of physical exercise or sports aimed at health? Do you engage in low levels of physical exercise aimed at health?
(4) Handgrip Strength (HS)	Clinical test: (Yes = 1, No = 0) Men: <26 kg, Women: <18 kg
(5) Walking Speed (SP)	Clinical test: (Yes = 1, No = 0) Speed < 1.0 m/s (obtained by five-meter walking test)

**Table 3:** Results of the statistical analysis on five gait parameters for the groups classified by the J-CHS.

Parameter	Group	Mean ± SD	Pairwise 95% CI *	Group *	*p*-Value	d-Value
SL	Frail	0.52 ± 0.17	0.40–0.64	F and PF	0.026	0.964
	Pre-Frail	0.61 ± 0.07	0.58–0.64	PF and NF	0.015	0.707
	Non-Frail	0.67 ± 0.09	0.64–0.70			
SC	Frail	1.88 ± 0.09	1.82–1.94	F and PF	<0.001	1.712
	Pre-Frail	2.15 ± 0.17	2.08–2.22	PF and NF	0.570	0.160
	Non-Frail	2.12 ± 0.11	2.08–2.16			
GV	Frail	0.97 ± 0.33	0.74–1.20	F and PF	<0.001	1.509
	Pre-Frail	1.32 ± 0.19	1.24–1.40	PF and NF	0.059	0.538
	Non-Frail	1.43 ± 0.21	1.35–1.51			
6MWD	Frail	253 ± 118	172–335	F and PF	<0.001	1.508
	Pre-Frail	385 ± 76	355–417	PF and NF	0.019	0.672
	Non-Frail	430 ± 60	409–453			
6MWEE	Frail	11.10 ± 5.01	7.31–14.88	F and PF	<0.001	1.705
	Pre-Frail	19.70 ± 4.58	17.75–21.66	PF and NF	0.171	0.388
	Non-Frail	21.48 ± 4.40	19.81–23.14			

* Note: CI = confidence interval; F = frail; PF = pre-frail; NF = non-frail.

**Table 4:** Correlation between five items of the J-CHS and parameters (SL and SC).

Parameter	SL				SC			
	No *	Yes *	*p*-Value	d-Value	No *	Yes *	*p*-Value	d-Value
WL	0.63 ± 0.11	0.61 ± 0.11	0.512	0.238	2.11 ± 0.15	2.03 ± 0.20	0.182	0.488
HS	0.65 ± 0.08	0.54 ± 0.13	<0.001	1.129	2.11 ± 0.14	2.07 ± 0.21	0.442	0.237
EX	0.63 ± 0.11	0.61 ± 0.09	0.469	0.228	2.13 ± 0.14	1.99 ± 0.17	0.004	0.952
SP	0.65 ± 0.08	0.47 ± 0.13	<0.001	1.725	2.13 ± 0.14	1.91 ± 0.14	<0.001	1.406
AC	0.65 ± 0.10	0.58 ± 0.12	0.037	0.610	2.11 ± 0.15	2.09 ± 0.18	0.666	0.125

* Note: No: the item is qualified; Yes: the item is not qualified.

## Data Availability

The data presented in this study are available on request from the corresponding author.

## Appendix A

The acronyms used in this manuscript are listed in Table A1.

**Table A1:** The acronyms used in the manuscript.

Symbol	Description
SL	Stride length
SC	Step cadence
J-CHS	Japanese version of Cardiovascular Health Study
TUG	Timed up and go
6MWT	Six-minute walking test
ATS	American thoracic society
6MWD	Six-minute walking distance
WMS	Walking muscle strength
6MWEE	Six-minute walking energy expenditure
WL	Weight loss
EX	Exhaustion
PA	Physical activity
HS	Handgrip strength
SP	5-m walking speed
GMS	Gait measurement system
IIR	Infinite impulse response
SW	Straight walk
UW	U-turn walk
GV	6MWT gait velocity
FFT	Fast Fourier Transform
BMR	Basal metabolic rate
MET	Metabolic equivalent of task
HBE	Harris–Benedict equation
ROC	Receiver operating characteristic
AUC	Area under the curve
ASWG	Asian sarcopenia working group
